# Study of the dairy characters of lactating Murrah buffaloes on the basis of body parts measurements

**DOI:** 10.14202/vetworld.2017.17-21

**Published:** 2017-01-09

**Authors:** Sandeep Dhillod, Dipankar Kar, C. S. Patil, Subhasish Sahu, Narender Singh

**Affiliations:** 1 Department of Livestock Production Management, College of Veterinary Sciences, Lala Lajpat Rai University of Veterinary and Animal Sciences, Hisar - 125 004, Haryana, India; 2 Department of Animal Genetics and Breeding, College of Veterinary Sciences, Lala Lajpat Rai University of Veterinary and Animal Sciences, Hisar - 125 004, Haryana, India

**Keywords:** body parts measurements, correlation, dairy characters, regression

## Abstract

**Aim:**

The aim of the study was to correlate the milk yield of Murrah buffaloes with certain body parts measurements.

**Materials and Methods:**

A total of 70 lactating Murrah buffaloes were selected from Buffalo Farm, Lala Lajpat Rai University of Veterinary and Animal Science, Hisar and were randomly selected in a range from first to fifth parity. Traits studied were 305 days milk yield (MY), body weight (BW), body length (BL), muzzle width (MW), height at wither (HW), abdominal girth (AG), chest girth (CG), body depth fore, body depth rear, hip bone distance (HBD), pin bone distance (PBD), skin thickness (STK), and tail length (TL). Data were collected and statically analyzed by Pearson’s correlation method.

**Result:**

The result of this study showed that Murrah buffaloes had the average 2604.8±39.5 kg for MY, 556.1±4.9 kg for BW, and 152.2±0.8 cm for BL. This study showed that buffaloes had positive significant (p<0.05) correlation between MY and BW (0.26). Highly significant (p<0.01) correlation was observed between MY and AG (0.64), MW (0.42). Significant (p<0.01) negative correlation was observed between MY and STK (−0.79). Different body part measurements (BW, BL, HW, AG, CG, MW, TL, BD, PBD, HBD, STK) were significantly correlated with each other.

**Conclusion:**

This study can be helpful as a selection tool to enhance and evaluate the production potential by setting standards of Murrah buffalo breed. BW, abdominal growth, muzzle thickness, and STK were found key factors while selecting a dairy Murrah buffalo.

## Introduction

Buffalo (*Bubalus bubalis*) is premier dairy animal of India and holds the greatest promise and potential for milk, meat, and draught. Of all the domestic animals, the Asian water buffalo holds the greatest promise and potential for production. The buffalo did not receive the attention it deserved in the post independent era, but the economic advantage has swayed the attention of the scientists as well as the policy makers toward this bovine Black Gold.

Murrah breed of buffalo, the pride of Haryana, is a milk type animal. The home tract of Murrah buffalo is Rohtak, Jind, Hisar and Bhiwani districts of Haryana. It is also found in Nabha and Patiala districts of Punjab and around Delhi. India has over 108 million heads of buffaloes, which is approximately 57% of total world buffalo population [1] contributing 50 million tons of milk, which accounts for 55% of total milk production (92 million tons) in India [2]. These figures show great superiority of buffalo in production ability. During the last 10 years, world buffalo population increased by approximately 18 million showing annual increase of about 1.13% which is mainly due to the increase in buffalo population in Asian countries. The percent increase in India was about 1.0% as compared to 1.09% in Asia and 2.58% in rest of the world [3].

This will also demand that our breed and species support should preferentially be for buffalo development [4]. The organized dairy sector in India is largely dependent on buffalo milk because of their contribution to total milk production, rich fat and total solid content [5]. Delivering the keynote address at the South Asian Dairy Congress, Dr. A. K. Srivastava, Director, National Dairy Research Institute, said that efforts must be made to reach the target of producing 191 million tons of milk a year by 2020, he said milk production was only 17.1 million tons when white revolution began, and now it is about 121.7 million tons [6].

Physical appearance of most of exotic cattle breeds in different parts of the world has been studied extensively but such type of information in Murrah buffaloes is scanty especially the concept of body parts measurements, and milk yield (MY) relationship in dairy buffaloes is very recent. Only some basic information in relation to body parts measurements is available.

The positive correlations between few body measurements and MY in different genotypes of Holstein cattle have been determined [7]. Exotic dairy animals have a significant relationship between body measurements of and milk production [8]. Therefore, it was considered imperative to undertake this study so that information on important body parts measurements for lactating Murrah breed can be established. Hence, the potential of the animal would appropriately be judged, and selection would be made in positive direction. The body structure of dairy buffaloes is not only important to demonstrate the beauty of dairy animals but also for their high milk productivity and the expected relationship with MY can be established.

## Materials and Methods

### Ethical approval

All the procedures have been conducted in accordance with the guidelines laid down by the Institutional Ethics Committee.

### Place of study

The study was conducted in the Department of Livestock Production Management, College of Veterinary Sciences, Lala Lajpat Rai University of Veterinary and Animal Sciences (LUVAS), Hisar.

### Body parts measurements

A total of 70 lactating Murrah buffaloes were selected from Buffalo Farm, Department of Livestock Production Management, LUVAS, Hisar. Buffaloes were randomly selected in a range from first to fifth parity on the basis of availability at farm. The various body parts measurements were recorded along with MY. Body length (BL) was measured as the distance from the point of shoulder to the point of pin bone. Chest girth (CG) was measured as the circumference of body over the chest of animal just behind the elbow. Abdominal girth (AG) recorded as the circumference of the body over the flank just in front of the udder. Skin thickness (STK) recorded with the help of vernier caliper over the side skin of the buffalo’s neck region. The reading was then divided by two to know the exact STK value of one-fold.

The animals were weighed on 1000 kg capacity “AVERY” weighing balance fitted in weighing platform for taking body weight (BW). The wither height was measured when the animal was in standing position evenly on the ground with normal posture. The muzzle width (MW) was measured with help of big horn caliper and full MW was recorded. The distance between two hip bones and pin bones was measured with help of measuring tape. Body depth was calculated by difference between the top line height and height at bottom line. Tail dimensions were measured with help of measuring tape.

### Statistical analysis

The data were analyzed by applying Pearson’s correlation coefficient tested by the standard method suggested by Snedecor and Cochran [9]. The quantity r, called the linear correlation coefficient, measures the strength and the direction of a linear relationship between two variables. The linear correlation coefficient is sometimes referred to as the Pearson product moment correlation coefficient in honor of its developer Karl Pearson. The mathematical formula for computing r is:


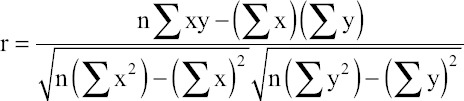


Where, n is the number of pairs of data.

## Results

The results of this study were found that the average 2604.8±39.5 kg MY in 305 days, 556.1±4.9 kg BW, 152.2±0.8 cm BL, 17.3±0.1 cm MW, 135.8±0.5 cm HW, 226.3±4.8 cm AG, 214.6±1.2 cm CG, 97.8±1.3 cm TL, 76.3±1.3 cm BD, 64.2±0.5 cm hip bone distance (HBD), 39.0±0.5 cm pin bone distance (PBD), and 1.6±0.0 cm STK in Murrah buffaloes (Table-1).

**Table-1:** Mean of different body parts measurements along with SEs.

Number of observation (N)	Body measurements	Mean±SE
70	MY (kg)	2604.77±39.47
70	BW (kg)	556.11±4.91
70	BL (cm)	152.23±0.83
70	MW (cm)	17.25±0.10
70	HW (cm)	135.78±0.46
70	AG (cm)	226.27±4.78
70	CG (cm)	214.57±1.17
70	TL (cm)	97.79±1.31
70	BDF (cm)	76.23±1.27
70	BDR (cm)	83.58±2.14
70	HBD (cm)	62.24±0.48
70	PBD (cm)	39.03±0.46
70	STK (mm)	8.0±0.02

MY=Milk yield, BW=Body weight, BL=Body length, MW=Muzzle width, HW=Height at wither, AG=Abdominal girth, CG=Chest girth, BDF=Body depth fore, BDR=Body depth rear, HBD=Hip bone distance, PBD=Pin bone distance, STK=Skin thickness, TL=Tail length

MY of animals showed significant (p<0.05) correlation with the BW (0.26) of animals and significant (p<0.01) and positive correlation between MY and AG (0.64), MW (0.42) was also observed (Table-2). STK (−0.79) showed negative and significant (p<0.01) correlation with MY. The significant (p<0.05) correlation was observed between BW (0.26) and MY. Likewise BL, MW (0.37 and 0.34) were significantly (p<0.01) correlated with BW. Positive and significant (p<0.01) correlation were found between AG, CG and HBD and BW. BW was significantly (p<0.01) correlated with AG (0.47) and CG (0.35). Moderate significant (p<0.05) correlation was observed between BW and rear body depth (BDR).

**Table-2:** Correlation between MY and body parts measurements in the Murrah buffaloes.

Trait	MY	BW	BL	MW	HW	AG	CG	TL	BDF	BDR	HBD	PBD	STK
MY	-	0.26*	0.17	0.42**	0.16	0.64**	0.14	−0.03	0.11	0.21	0.03	0.02	−0.79**
BW	0.26*	-	0.37**	0.34**	0.21	0.47**	0.35**	0.03	0.12	0.28*	0.41**	0.13	−0.31**
BL	0.17	0.37**	-	0.17	0.18	0.23*	0.24*	0.19	0.01	0.33**	0.44*	0.10	−0.28*
MW	0.42**	0.34**	0.17	-	0.18	0.57**	0.13	0.08	0.10	0.20	0.05	0.04	−0.66**
LJW	0.13	0.31**	0.17	0.12	0.13	0.15	0.16	−0.25*	0.07	0.01	0.22*	0.02	−0.16
HW	0.16	0.21	0.18	0.18	-	0.15	0.42**	−0.06	0.22	0.47**	0.37**	0.06	−0.12
HCG	−0.05	−0.05	−0.13	−0.01	0.52**	−0.15	−0.07	−0.02	−0.04	−0.50**	0.19	0.17	0.06
AG	0.64**	0.47**	0.23*	0.57**	0.15	-	0.36**	0.19	0.06	0.60**	0.26**	0.07	−0.60**
CG	0.14	0.35**	0.24*	0.13	0.42**	0.36**	-	−0.10	0.17	0.36**	0.33**	0.12	−0.14
TL	−0.03	0.03	0.19	0.08	−0.06	0.19	−0.10	-	−0.20	0.19	0.14	0.07	0.02
TSL	0.04	0.18	0.13	0.04	−0.06	0.19	−0.12	0.57**	−0.03	0.20	0.03	−0.06	−0.05
WSL	0.11	0.08	0.19	0.09	0.01	0.20	0.00	0.51**	−0.43**	0.06	0.03	0.26*	−0.15
BDF	0.11	0.12	0.01	0.10	0.22	0.06	0.17	−0.20	-	0.27*	0.06	0.18	0.19
BDR	0.21	0.28*	0.33**	0.20	0.47**	0.60**	0.36**	0.19	0.27*	-	0.54**	0.61**	−0.19
HBD	0.03	0.41**	0.44**	0.05	0.37**	0.26*	0.33**	0.14	0.06	0.54**	-	0.33**	−0.15
PBD	0.02	0.13	0.10	0.04	0.06	0.07	0.12	0.07	0.18	0.10	0.33**	-	0.02
STK	−0.79**	−0.31**	0.28*	−0.66**	−0.12	−0.60**	−0.14	0.02	0.19	−0.19	−0.15	0.02	-

* p<0.05,

** p<0.01. MY=Milk yield, BW=Body weight, BL=Body length, MW=Muzzle width, HW=Height at wither, AG=Abdominal girth, CG=Chest girth, BDF=Body depth fore, BDR=Body depth rear, HBD=Hip bone distance, PBD=Pin bone distance, STK=Skin thickness, TL=Tail length, HCG=Height at chest girth, TSL=Tail switch length, WSL=White switch length

BL was significantly (p<0.01) correlated with BW, CG (p<0.05) and HBD (0.37, 0.24 and 0.44) and was found to have no significant correlation with other body measurements. Significant (p<0.01) and positive correlation were found between MW and MY, BW and AG (0.42, 0.34 and 0.57). Height at wither (HW) was positively and significantly (p<0.01) correlated with CG (0.42), BDR (0.47) and HBD (0.37). AG was significantly (p<0.01) correlated with MY (0.64), BW (0.47), MW (0.57), CG (0.36), BL (0.23), BDR (0.31), and HBD (0.26) but negative and significant correlation with STK (−0.60).

Significant (p<0.01) correlation were observed between CG and BW, HW, AG, BDR and HBD (0.35, 0.42, 0.36, 0.35 and 0.33), respectively. Significant (p<0.01) correlation was found between BDR and BL, HW, AG and CG (0.33-0.60). Moderate significant (p<0.05) correlation was observed with HW (0.28) and fore body depth (0.27). Highly significant (p<0.01) correlation was found between HBD and BW, BL, wither height, CG and PBD (0.41, 0.44, 0.37, 0.33 and 0.33), respectively. Highly significant and negative correlation (p<0.01) were found between STK and MY, BW, MW and AG (−0.80, −0.31, −0.66 and −60). While significant coefficients of correlation (p<0.05) were found between STK and BL (0.28).

## Discussion

The 305-day lactation yield was positively and significantly correlated with live weight as well as with most of physical traits [10]. A negative and significant (p<0.01) correlation between MY and STK (−0.79) was observed in the study. Milk was higher in buffaloes having thin skin than medium. The thick skin and MY were significantly higher in buffaloes having thin skin in CIRB, Hisar [11]. Animal with thin skin could dissipate more heat and thus be more efficient for the production of milk in warm regions [12]. However, some reported positive and significant correlation of STK with MY in Hariana cattle [13]. The present results were also not in agreement with the study who convey that BL can be an indicator to predict daily MY of Kenana cattle [14].

The relationship of BL with heart girth, pin to PBD and hook to hook bone distance was found highly positive and significant and same with milk production [15].

The live weight of animals has strong association with MY [10]. STK showed negative correlation with BW, BW and BL, MW, lower jaw width, AG, CG, HBD and negative correlation with STK which fell in the various study [10,16]. MY was significantly (p<0.01) and positively correlated with MW and in agreement with study done in LUVAS [17]. MW was found to be strongly associated with BW, AG but significant negative correlation with STK.

The study revealed that AG (0.64) was positively and significantly (p<0.01) correlated with MY and the present study was in agreement with previous studies [12,18] who reported that milk production increases by 7.8 lb per increase of 1″ over of 83.54″ AG in Murrah buffaloes. The different body measurements (HG, AG) were significantly and positively correlated with each other in crossbred cows and Murrah buffaloes [16].

The study revealed that BL had positive, but no significant correlation with MY but have significant correlation with BW, CG, and HBD but negative and significant correlation with STK [19]. The study revealed that MY was not significantly associated with HW and height at chest from ground and study was not found in agreement with Lin *et al*. [7] reported that milk production traits were all positively correlated with body measurements. The study revealed that milk was not associated with CG or heart girth. This study revealed that body depth had positive correlation with MY but not found to have significant correlation. The study revealed that HBD was not associated significantly with MY but on regression with MY was done HBD was found associated with MY. The study revealed that PBD was not associated significantly with MY. The relationship of pin to PBD was found positive and significant correlation with milk production in Nili Ravi buffaloes [15].

The study depicted that on the basis of data tail length (TL), tail switch length and white switch length not found to associate with MY and similar findings were also observed [12]. However, TL had no significant correlation with MY and similar findings were also observed [19]. It was also observed that TL was not correlated with MY in Nili-Ravi buffaloes [20]. This study advocates that TL plays no role in milk production.

## Conclusion

The study revealed that body part measurements such as BW, AG, MW, and STK have significant effect on MY in Murrah buffaloes. Some of the other body measurements, *viz*., BL, lower jaw width, CG, HW, and body depth have been found positively correlated with MY, but these relationships need further investigation so that selection can be made on the basis of these traits. Hence, the body part measurements should be utilized for selection of buffaloes for milk production. The work is very important for selection and judging of Murrah buffaloes and further research can be carried in this field to create prediction equations through multiple regression analysis for betterment of results.

## Authors’ Contributions

SD, DK designed the work. SD conducted the research work. Data analysis and manuscript was written by SD, SS and CSP under the guidance of DK. NS involved in recording observations and critical revision of manuscript. All the authors have read and approved the final manuscript.
